# Metal Content and Structure of Textiles in Textile Metal Threads in Croatia from 17th to 20th Century

**DOI:** 10.3390/ma15010251

**Published:** 2021-12-29

**Authors:** Kristina Šimić, Ivo Soljačić, Domagoj Mudronja, Tihana Petrović Leš

**Affiliations:** 1 Faculty of Textile Technology, University of Zagreb, 10000 Zagreb, Croatia; isoljac@ttf.unizg.hr; 2 Natural Science Laboratory Institute, Croatian Conservation, 10000 Zagreb, Croatia; dmudronja@hrz.hr; 3 Faculty of Humanities and Social Sciences, University of Zagreb, 10000 Zagreb, Croatia; tihana.petrovic-les@hi.t-com.hr

**Keywords:** textile metal threads, Croatian historical textiles, analysis, SEM-EDX, cross-section

## Abstract

Textile metal threads were used to decorate historical Croatian textiles. There are three basic types of metal threads usually used on historical textiles in Croatia. These are narrow stripes, wires, and combined metal textile yarn called “srma”, made of metal thread spirally wrapped around the nonmetal textile yarn. Textile yarns were made of silk, linen, wool, or cotton. Metal threads were primarily made of gold, silver, and copper, and different alloys of these metals or threads are layered in the structure. Analysis of metal threads with three different methods was made and the most adequate method for the analysis of metal threads from historical textiles was established. Metal thread analysis was performed with scanning electron microscopy with an energy-dispersive X-ray detector (SEM-EDX), which was determined to be the most suitable for the analysis of historical textiles if cross-section analysis of metal threads is also performed. Textile threads from combined metal textile threads were analysed with a light microscope. This information of the metal threads’ content and structure as well as the composition of textile thread can lead to an understanding of the technology of production threads and also temporal and spatial dating of textile objects which is helpful to conservators and restorers of valuable historical textiles.

## 1. Introduction

Precious metals have been used to decorate textiles since ancient times to create luxury items for the secular and religious elite. The topic of this paper is the analysis of metal threads and textile yarns wrapped with metal threads from Croatian historical textiles. The term historical textiles includes clothing of the peasant, civil, and aristocratic classes, and liturgical textiles from the period from the 17th to the first half of the 20th century [1,2]. Conservators and restorers have been engaged in metal threads analysis before processing an individual object [3]. The content of metals in metal threads determines their properties, purpose, and origin, so physicochemical analysis is a very important step in their characterization. The analysis of metal threads in historical textiles also enables the correct choice of methods for cleaning, conservation, and restoration of old, historically very valuable textile materials [4]. Valuable historical textiles decorated with metal threads are found on liturgical vestments and folk costumes and are kept in museums and church treasuries throughout Croatia. The purpose of metal threads in textiles since the beginning of their appearance has been to show power, wealth, and honour. In ceremonial folk costumes, it also signified the position of the owner in society, while in liturgical vestments it served the glory of God [5]. More recently, metal threads have some other roles such as strengthening the material and removing static electricity or electromagnetic radiation and they appear on some other textile items [6]. For the purpose of this paper, only older threads from the period from the 17th to the mid 20th century were analysed. Thread collection was carried out from all over Croatia from all of its different regions.

In Croatia, garments have been decorated with metal threads since the Middle Ages (the period of the Old Croatian state) as evidenced by the findings of reticulated creations in graves. These remains speak of the early use as well as of the trade of the Kingdom of Croatia within the wider Mediterranean area [7]. Textiles decorated with various types of metal threads were used until the modern period on clothing and other textiles, primarily for liturgical use, then on the clothes of the civil and aristocratic class, ceremonial folk costumes, and flags [6]. Shiny metal threads in weaving or embroidery are found on women’s and men’s costumes of the Adriatic, Dinaric, and mostly Pannonian ethnographic zone, but only in the area of eastern Croatia. Eastern Croatia is best known for its gold embroidery technique; the gold embroidery of the Županja, Vinkovci, Brodsko Posavlje, and the particularly prominent gold embroidery of the Đakovo region are different [8]. Craft, tailored embroidery, originating from Turkey, is found in women’s and men’s folk costumes of the Adriatic and Dinaric zones. It is a fine embroidery made of gold, silver, and silk ribbons on silk, velvet, and wool fabrics [9]. The application of embroideries with gold and silver threads is still used in the renewal of costumes, decoration, and production of new fashionable clothing, liturgical vestments, on military uniforms for marking ranks, as well as on decorative fabrics [6].

Analyses of metal threads in Croatia are determined in certain historical textiles mostly by thin-layer chromatography, inductively coupled plasma-optical emission spectrometry (ICP-OES) and using atomic absorption spectrometry (AAS). These methods are destructive and require the dissolution of samples prior to analysis. These destructive methods require special and long-term sample preparation. The results give a precise chemical composition, but do not provide an overview of the sample’s structure [10,11]. The most prominent authors who have dealt with the history of origin, but also with the analysis of metal threads in the world and published many papers are Márta Járó [12,13,14] from Hungary and Anna Karatzani [15] from Greece. The composition of metal threads from textiles is most often determined according to the data in the world literature by non-destructive methods Scanning Electron Microscopy with Energy Dispersive X-ray Spectroscopy (SEM-EDX), X-ray Fluorescence Spectroscopy (XRF), and Particle Induced X-ray Spectroscopy (PIXE) [16,17,18,19,20,21,22]. In our previous research [23] XRF, PIXE, and SEM-EDX were used. It was determined that XRF is most suitable for the fast selection of samples while PIXE and SEM/EDX are more suitable for quantitative analysis. There are differences in penetration depth of X-rays (100–200 µm), electrons from SEM-EDX (0.5 µm), and 2 MeV protons (20 µm in metals) for PIXE analysis. For homogeneous samples, different depth penetration was not a problem and the results were similar, but for samples of metal threads with a layered structure such as gilded and silver-plated samples, results were different [23].

Therefore, the SEM-EDX method was selected as most acceptable and suitable for the analysis of metal threads from historical textiles but it had to be used both in surface and cross-section analysis. This method is fast and simple and with minimal sample preparation, it reveals the morphology of the sample by SEM analysis, while the results of the EDX spectrum detect the chemical composition, but also the type and range of corrosion products found on the metal surface [24,25,26].

## 2. Materials and Methods

**Figure 1:**
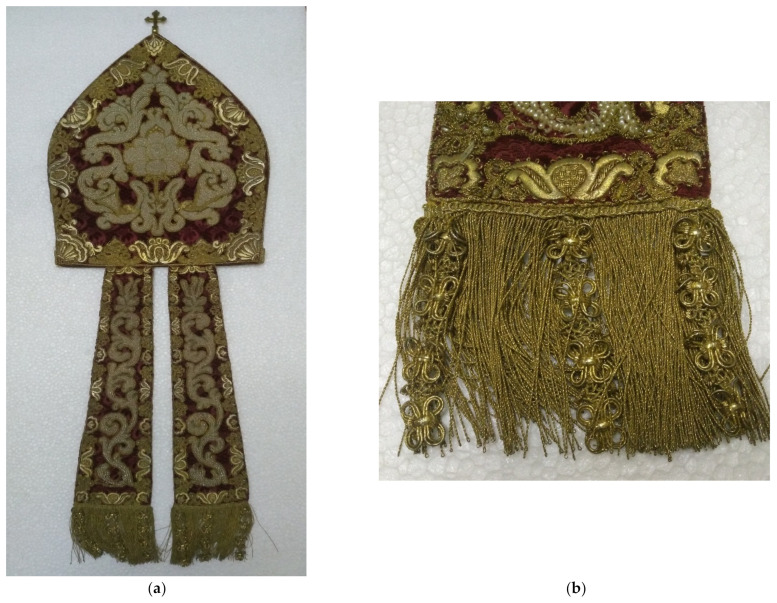
Miter from Treasury of the Zagreb Cathedral, work of Petretić’s workshop (1648–1667): (**a**) Miter; (**b**) detail of the miter, fringes.

**Figure 2:**
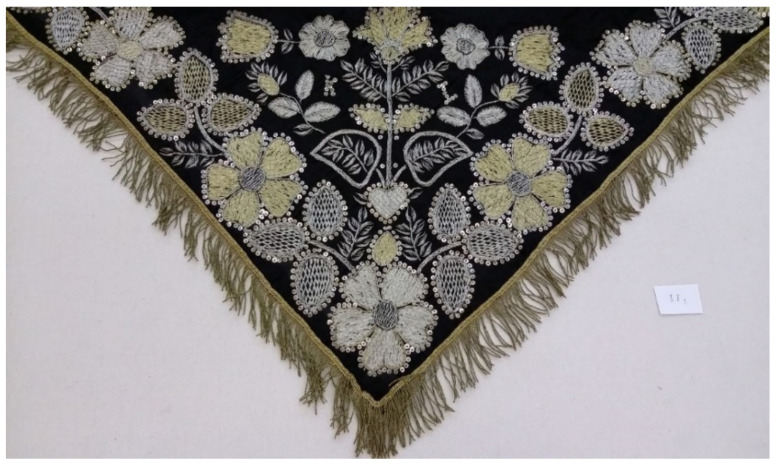
Scarf from the Ethnographic Museum Zagreb, work of Đakovo region, end of the 19th to the middle of the 20th century.

Samples of metal threads were collected with the permission and supervision of conservators and restorers so that only hanging threads were taken for analysis. All regions of Croatia are included: Osijek east, Varaždin north, Zagreb, Prilišće in central Croatia, Novigrad near Zadar, Split, and Dubrovnik in southern Croatia. Two groups of samples from metal thread stand out and these are from liturgical vestments and threads from folk costumes. Folk costumes unlike liturgical vestments often have some local characteristics related to the customs of that area and have special features. Liturgical vestments samples are from the Treasury of the Zagreb Cathedral, Varaždin City Museum, Prilišće Museum, Novigrad Museum, and Slavonia Museum in Osijek. Folk costumes samples are from Ethnographic Museum Zagreb, Museum in Sinj, Ethnographic Museum Split, Ethnographic Museum Dubrovnik, and Slavonia Museum in Osijek. Figure 1 shows a miter (liturgical hat) abundantly decorated with metal threads from the Treasury of the Zagreb Cathedral, while Figure 2 shows a scarf (part of a folk costume) from the Ethnographic Museum Zagreb.

**Figure 3:**
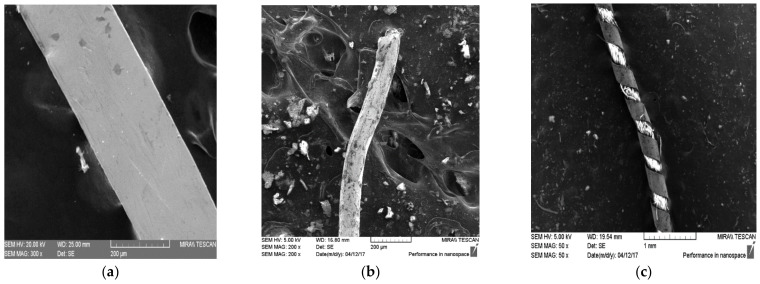
Three different metal threads found on historical textiles in Croatia investigated by SEM-EDX: (**a**) metal narrow stripe; (**b**) metal wire; (**c**) combined metal textile threads called “srma”.

Threads that are found on these historical items can be classified into three groups (Figure 3): (**a**) metal narrow stripes or lamellas; (**b**) metal wires, and (**c**) combined metal textile threads called “srma”.

Metal threads can be woven into textiles, part of a fringe hanging from textiles, but also it can be in the decorative ribbons stitched on textiles. There are 156 samples of metal threads, 73 are from liturgical vestments (Table 1) and 83 from folk costumes (Table 2). Most of the collected samples are combined metal textile threads (114), and the least are wires (16), while 26 are narrow stripes.

Analysis of surface and cross-section of 156 samples was performed with SEM-EDX. Characteristics of used SEM-EDX device (Tescan, Brno, Czech Republic); device type: MIRA FE-SEM, electron gun: Schottky emitter, operating voltage: 20 kV, electron flux current: 2 pA to 100 nA, working distance: 25 mm, detector: Bruker AXS, Quantax EDX detector type SDD (Silicon Drift Detector). Textile yarns from combined metal textile threads were analysed with a light microscope. The Olympus cx22 microscope with a total magnification of 100× was used for analysis.

**Figure 4:**
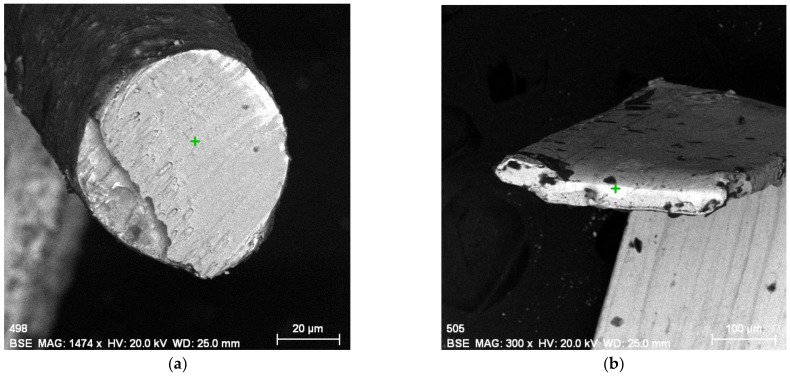
SEM-EDX pictures of cross-sections: (**a**) silver-plated wire from Split folk costume; (**b**) gilded narrow stripe from Zagreb Cathedral liturgical vestments.

During the analysis, it was found that the cross-section of metal threads can be obtained easily by bending the thread at an angle of 90°. The metal thread was cut to a suitable length for measurement. It is placed at a right angle so that the cut tip (part) remains open towards the direction from which the electron beam is coming. In this way, the cross-section is visible as the surface that the SEM device scans. Due to the stiffness of the metal thread, it remains in a bent position, so there is no need to fix the thread with resin, no coatings on the samples were done. SEM-EDX can determine the metal composition inside the thread in a bent position (Figure 4). A green + in the figure indicates the place where the metal composition is determined. There is also a different colour of the thread in the interior (darker grey) and on the edges, which is the surface (lighter grey), so it can be concluded that it is a different type of metal, which was confirmed by analysis. The metal on the surface is lighter-coloured, as can be seen from the picture, is not evenly distributed over the entire surface of the thread.

## 3. Results

SEM-EDX pictures (Figure 4) and analysis of the cross-section confirmed which samples are homogeneous and which have layered structure, gilded or silver-plated. Figure 4 shows silver-plated copper metal wire from a Split folk costume and gilded silver metal narrow stripe from Zagreb Cathedral liturgical vestments. Differences between the composition of the metal threads from the liturgical vestments and folk costumes were observed.

**Figure 5:**
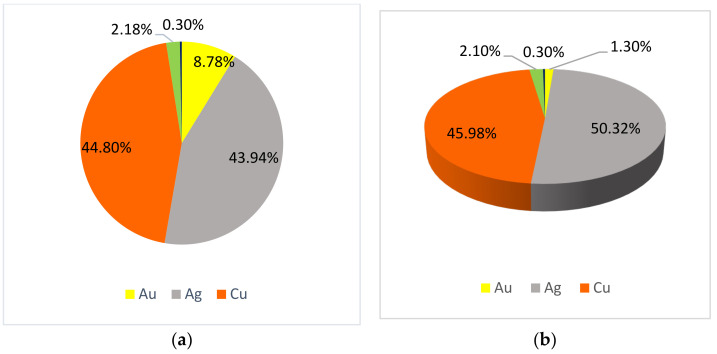
Metal content in liturgical vestments: (**a**) on the surface (**b**) in cross-section.

**Figure 6:**
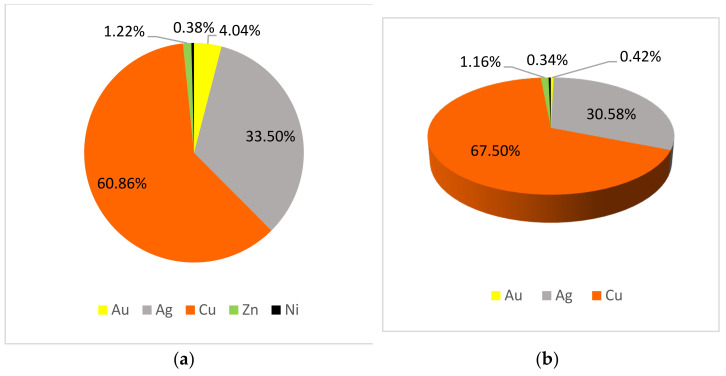
Metal content in folk costumes: (**a**) on the surface (**b**) in cross-section.

The main difference between the chemical composition of metal threads from folk costumes and those from liturgical vestments is that in some threads from folk costumes nickel appears, and in some threads from liturgical vestments aluminium appears (Figure 5 and Figure 6). However, these elements are minimally present in alloy samples with less than 0.50%.

**Figure 7:**
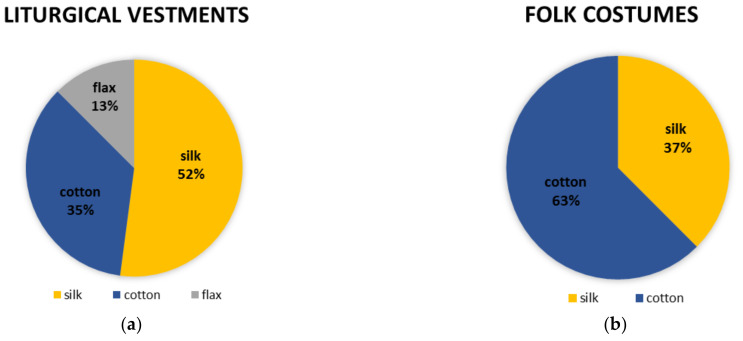
Compared textile yarns in combined metal textile threads (**a**) from liturgical vestments (**b**) from folk costumes.

The main difference in comparing textile yarns from combined metal textile threads from liturgical vestments and those from folk costumes is that flax is present in some threads from liturgical vestments (Figure 7). Textile yarns from combined metal textile threads from folk from costumes are only silk or cotton.

Silk is more represented in textile yarns from liturgical vestments than in textile yarns from folk costumes and only one in metal threads from the Treasury of the Zagreb Cathedral. Metal threads with silk core are of better quality and more valuable. Most textile threads from folk costumes are made of cotton which are lower quality threads [14].

The textile part of combined metal textile threads is matched with the metal part in quality and colour. In samples with metal threads of gilded silver or pure silver, there is a silk textile core. Cotton and linen are textile parts of samples that have copper or copper alloys for the metal component. The gold metal threads contain yellow textile cores, while the silver ones contain a white textile core. All this indicates the dedication and attention to detail in the production of the metal threads used to decorate historical textiles.

Table 3 and Table 4 show in detail which types of metal threads in which quantities were found in different places in Croatia. (The eleven gilded copper samples from Zagreb folk costumes are divided into two different groups. The first is five samples of copper gilded with gold-silver alloy and the second is six samples of gilded silver-copper alloy).

## 4. Discussion

The liturgical vestment metal threads from the Treasury of the Zagreb Cathedral stand out in particular: they are the oldest from the 17th and 18th centuries and most valuable specimens of gilded silver and pure silver. The only textile yarn in combined metal textile threads is silk. These are items decorated by embroiderer Stoll, metal threads that he procured himself, and items donated by the empress Maria Theresa. Only the best materials, including metal threads, were preserved in the treasury [6]. So the results from this sample belong to a special group and are the most valuable.

From folk costumes samples, the samples from Sinj stand out. They are the most valuable because silver dominates at about 60%, there is gold only on the surface of about 8%, and there is also copper in 30–40%. In the combined textile metal thread samples from Sinj, silk dominates as a non-metallic core, only two samples are made of cotton that have copper metal threads. There is no documentation of age for these samples but it is assumed that they are older ones.

Liturgical and folk metal threads from Osijek are mostly made of pure copper and some of copper–zinc and also silver-plated copper and are from the 19th and 20th centuries. Gold is present only on the surface on two samples, averaging 3.8% in folk costume metal threads and three samples average 11% in liturgical vestment threads. It is observed that older metal threads from the 17th and 18th centuries are better quality with more gold and silver than those of the 19th and 20th centuries as stated in the paper by Karatzani [15].

It is observed that there is more gold and silver on the threads of the liturgical vestment on the surface and in the cross-section, Figure 5 shows that accordingly, there is less copper. Copper and silver are the most common elements on metal threads. Zinc is found only on some threads, in ranges from 1 to 3%. A bit more Zn was present on metal threads from the liturgical vestments which are more valuable than those from folk costumes because they are older, especially those from the Treasury of the Zagreb Cathedral. The value of the thread was to show the power and grace of the vestments for liturgical celebrations. This is to be expected because Holy Mass in the Catholic Church is considered the most solemn act dedicated to God, and the priest should be solemnly dressed. This is true regardless of the place and time when the attire was applied. An analysis of samples from museums in Varaždin, Prilišće, Novigrad, and Osijek, which mostly keep vestments from their parishes, shows that liturgical vestments are richly decorated with valuable metal threads throughout Croatia in all churches and parishes, not just large cathedrals.

Metal threads were used as an ornament on festive folk costumes that were worn only on special occasions in women’s as well as men’s clothing [5]. Exceptionally, metal threads are woven into aprons that were used daily in Slavonia until the end of the 19th century. From visits to museums in Zagreb, Split, Dubrovnik, and Osijek and an insight into their material, it is evident that metal threads were mostly used as decoration in Slavonia, eastern Croatia, and in Dalmatia, the Dalmatian hinterland, and the Dubrovnik littoral. Metal threads were also used as decoration on clothes worn on the occasion of some traditional ceremonies, for which the particularly magnificent clothes for the Sinjska alka ceremony are an example.

## 5. Conclusions

Decoration of historic textiles with metal threads is very important in the cultural and religious life of Croatia and it reaches far into the past. In order to preserve this valuable historical textile from deterioration, its restoration and conservation are needed. Prior to each restoration procedure, a quick and easy detection of the used metal thread type is required. In this paper, the SEM-EDX method is presented as the most suitable for fast, simple, but also thorough analysis for this type of sample if a cross-section analysis is also performed. A simplified cross-sectional analysis of a metal thread is also presented in which, due to the stiffness of the metal, it is not necessary to place samples in the resin but only to bend the thread. By analysing the cross-section as well as the cross-sectional image with SEM-EDX, it is possible to determine whether samples are homogeneous or gilded and silver-plated.

Knowing the type and structure of the metal thread, the approximate origin of the metal threads for unknown samples can be determined. The analysis confirmed that older threads are more valuable because they have more gold and silver than newer ones.

Copper is the main element in metal threads from folk costumes but silver and copper are in metal threads from liturgical vestments. There is no copper on metal threads from Zagreb Cathedral liturgical vestments, only silver and gold. More gold and silver is found on metal threads from liturgical vestments than from folk costumes. The textile part of combined metal textile threads is matched with the metal part, so more silk is found on samples from liturgical vestments. Metal threads from liturgical vestments are more valuable than those from folk costumes because they are older, especially those from the Treasury of the Zagreb Cathedral.

## Figures and Tables

**Table 1:** Table of all metal thread samples from liturgical vestments.

Liturgical Vestments					
Metal Threads	Zagreb Cathedral	Varaždin	Prilišće	Novigrad	Osijek
Narrow stripes	3	7	3	4	1
Wires	-	1	2	-	2
Combined metal textile threads	11	10	11	12	6
Total	14	18	16	16	9

**Table 2:** Table of all metal thread samples from folk costumes.

Folk Costumes					
Metal Threads	Zagreb	Sinj	Split	Dubrovnik	Osijek
Narrow stripes	-	2	5	-	1
Wires	6	-	3	-	2
Combined metal textile threads	20	6	8	18	12
Total	26	8	16	18	15

**Table 3:** Table of all metal thread samples from liturgical vestments.

Liturgical Vestments					
Metal Threads	Zagreb Cathedral	Varaždin	Prilišće	Novigrad	Osijek
Silver	3	1	1	-	1
Gilded silver	11	5	4	2	3
Silver-plated copper	-	3	3	-	1
Copper	-	6	2	5	1
Gilded copper	-	-	1	-	-
Gold–silver alloy	-	2	-	2 and (1 Au/Ag/Al alloy)	-
Silver–copper alloy	-	-	-	6	-
Copper–zinc alloy	-	1	5	-	3

**Table 4:** Table of all metal thread samples from folk costumes.

Folk Costumes					
Metal Threads	Zagreb	Sinj	Split	Dubrovnik	Osijek
Silver	-	2	3	1	-
Gilded silver	-	3	3	2	-
Silver plated copper	10	2	2	3	2
Copper	3	1	2	-	6
Gilded copper	11	-	-	-	2 Copper gilded with Au/Ag alloy
Gold–silver alloy	-	-	4	1	-
Silver–copper alloy	-	-	-	7	-
Copper–zinc alloy	2	-	2	2 and (2 Copper–nickel alloy)	5

## References

[1] Raffaelli D., Čunko R., Dragičević M. (1982). Research of metal threads from the soil of Dalmatia at an interval of a thousand years. Textile.

[2] Soljačić I., Čunko R. (1994). Croatian textiles through history. Textile.

[3] Budicin M., Lucić Vujičić S. (2019). Renaissance golden velvet chalice from the Croatian Cathedral. Portal.

[4] Ahmed H.E. (2014). A new approach to the conservation of metallic embroidery threads in historic textile objects from private collections. Int. J. Conserv. Sci..

[5] Šimić K., Mudronja D., Pušić T., Soljačić I., Dragčević Z. (2016). Analysis of metal threads on historical Croatian textiles. Proceedings of the 8th International Textile, Clothing & Design Conference.

[6] Šimić K., Soljačić I., Pušić T. (2013). Application of metal threads in Croatia from the 11th to the 20th century. Textile.

[7] Dragičević M. (1981). Four fragments of textiles from old Croatian graves. Old Croat. Educ..

[8] Juzbašić J. (2018). Slavonian Gold Embroidery.

[9] Muraj A. (2001). Croatian Folk Costumes.

[10] Rezić I., Steffan I. (2007). ICP-OES determination of metals present in textile materials. Microchem. J..

[11] Rezić I., Spehar M., Jakovljević S. (2017). Characterization of Ag and Au nanolayers on Cu alloys by TLC, SEM-EDS, and ICP-OES. Mater. Corros..

[12] Járó M., Tamás G., Tóth A. (2000). The characterization and deterioration of modern metallic threads. Stud. Conserv..

[13] Járó M. (1990). Gold embroidery and fabrics in Europe XI–XIV centuries. Gold Bull..

[14] Járó M., Tóth A.L. (1991). Scientific identification of European metal thread manufacturing techniques of the 17–19th century. Endeav. New Ser..

[15] Karatzani A. (2007). The Evolution of a Craft: The use of metal threads in the decoration of late and post Byzantine ecclesiastical textiles. Ph.D. Thesis.

[16] Hložek M., Trojek T., Prokeš R., Linhart V. (2019). Mediaeval metal threads and their identification using micro-XRF scanning, confocal XRF, and X-ray micro-radiography. Radiat. Phys. Chem..

[17] Yurdun T., Karsli-Ceppioglu S., Oraltay R.G. (2012). Investigation of Metal Wired Coloured Historical Textile Using Scanning Electron Microscopy and HPLC-DAD. J. Chem. Chem. Eng..

[18] Tronner K., Nord A.G., Sjostedt J., Hydman H. (2002). Extremely thin gold layers on gilded silver threads. Stud. Conserv..

[19] Enguita O., Climent-Font A., Garcia G., Montero I., Fedi M.E., Chiari M., Lucarelli F. (2002). Characterization of metal threads using different PIXE analysis. Nucl. Instrum. Methods Phys. Res. Sect. B Beam Interact. Mater. At..

[20] Ahmed H.E. (2013). Identification and conservation of a rare Islamic textile decorated with metallic yarns. Egypt. J. Archaeol. Restor. Stud..

[21] Hacke A.-M., Carr C.M., Brown A., Howell D. (2003). Investigation into the nature of metal threads in a Renaissance tapestry and the cleaning of tarnished silver by UV/Ozone (UVO) treatment. J. Mater. Sci..

[22] Skals I. (1991). Metal thread with animal-hair core. Stud. Conserv..

[23] Šimić K., Zamboni I., Fazinić S., Mudronja D., Sović L., Gouasmia S., Soljačić I. (2018). Comparative analysis of textile metal threads from liturgical vestments and folk costumes in Croatia. Nucl. Instrum. Methods Phys. Res. Sect. B Beam Interact. Mater. At..

[24] Rezić I., Ćurković L., Ujević M. (2010). Simple methods for characterization of metals in historical textile threads. Talanta.

[25] Abdel-Kareem O., Harith M.A. (2008). Evaluating the use of laser radiation in cleaning of copper embroidery threads on archaeological Egyptian textiles. Appl. Surf. Sci..

[26] Duran A., Perez-Maqueda R., Perez-Rodriguez J.L. (2019). Degradation processes of historic metal threads used in some Spanish and Portuguese ornamentation pieces. J. Cult. Herit..

